# Health effects of residential wood smoke particles: the importance of combustion conditions and physicochemical particle properties

**DOI:** 10.1186/1743-8977-6-29

**Published:** 2009-11-06

**Authors:** Anette Kocbach Bølling, Joakim Pagels, Karl Espen Yttri, Lars Barregard, Gerd Sallsten, Per E Schwarze, Christoffer Boman

**Affiliations:** 1 Division of Environmental Medicine, Norwegian Institute of Public Health, Oslo, Norway; 2 Division of Ergonomics & Aerosol Technology (EAT), Lund University, Lund, Sweden; 3 Department of Atmospheric and Climate Research, Norwegian Institute for Air Research, Kjeller, Norway; 4 Department of Occupational and Environmental Medicine, Sahlgrenska University Hospital and Academy, University of Gothenburg, Gothenburg, Sweden; 5 Energy Technology and Thermal Process Chemistry, Umeå University, Umeå, Sweden

## Abstract

**Background:**

Residential wood combustion is now recognized as a major particle source in many developed countries, and the number of studies investigating the negative health effects associated with wood smoke exposure is currently increasing. The combustion appliances in use today provide highly variable combustion conditions resulting in large variations in the physicochemical characteristics of the emitted particles. These differences in physicochemical properties are likely to influence the biological effects induced by the wood smoke particles.

**Outline:**

The focus of this review is to discuss the present knowledge on physicochemical properties of wood smoke particles from different combustion conditions in relation to wood smoke-induced health effects. In addition, the human wood smoke exposure in developed countries is explored in order to identify the particle characteristics that are relevant for experimental studies of wood smoke-induced health effects. Finally, recent experimental studies regarding wood smoke exposure are discussed with respect to the applied combustion conditions and particle properties.

**Conclusion:**

Overall, the reviewed literature regarding the physicochemical properties of wood smoke particles provides a relatively clear picture of how these properties vary with the combustion conditions, whereas particle emissions from specific classes of combustion appliances are less well characterised. The major gaps in knowledge concern; (i) characterisation of the atmospheric transformations of wood smoke particles, (ii) characterisation of the physicochemical properties of wood smoke particles in ambient and indoor environments, and (iii) identification of the physicochemical properties that influence the biological effects of wood smoke particles.

## Background

Exposure to ambient particulate matter (PM) has been associated with a range of negative health effects, including increased morbidity and mortality from pulmonary and cardiovascular diseases [1-3]. Although residential wood combustion is a major source of particulate air pollution in many countries, relatively few studies have been performed to investigate the health effects associated with wood smoke exposure. The two most recent reviews on the topic concluded that the adverse health effects associated with wood smoke exposure in developed countries do not seem to be weaker than for ambient particles from other sources [4,5]. However, the reviewed literature suggested that the respiratory effects of wood smoke may be somewhat larger than the cardiovascular effects [5]. The use of wood or charcoal for heating or cooking during female adolescence was recently associated with chronic obstructive pulmonary disease later in life [6], providing further support for an association between wood smoke exposure and negative respiratory effects. In addition, a human inhalation study reported that wood smoke exposure affected both systemic and lung biomarkers, suggesting a potential impact of wood smoke particles also for cardiovascular diseases [7,8]. Recently, the International Agency for Research on Cancer (IARC) classified indoor emissions from household combustion of biomass fuel (mainly wood) as probably carcinogenic to humans (group 2A) [9].

The term residential wood smoke comprises emissions from a variety of biomass combustion appliances, such as open fireplaces, wood and pellet stoves, masonry heaters, and boilers for wood, wood chips and pellets [10-12] (see Additional file 1 for a brief description of the different types of combustion appliances). The combustion technology and air supply varies considerably between these different appliances, but also between old and new models of each type of appliance. In addition, the fuel type (e.g. wood logs, wood chips and pellets) and the condition of the fuel (e.g. moisture content and log size) also influence the efficiency of the combustion [11,13,14]. The physicochemical properties of particles emitted from residential biomass combustion differ considerably with combustion conditions and between combustion appliances [13,15]. Since epidemiological and experimental studies provide increasing evidence for the importance of physicochemical characteristics in the particle-induced biological effects [16,17], the differences in the physicochemical properties of particles originating from varying combustion conditions may influence their potential to induce biological effects.

Exposure to ambient PM in general has been associated with a range of pulmonary effects, such as decreased lung development and function, exacerbation of asthma, allergy, chronic obstructive pulmonary disease (COPD), pulmonary fibrosis and increased risk of lung cancer (reviewed in [3,18,19]). The cardiovascular diseases associated with particle exposure include atherosclerosis, myocardial infarction and stroke [20,21]. Several mechanisms, including particle-induced oxidative stress, inflammation, cytotoxicity and genotoxicity, have been proposed to explain the associations between particle exposure and adverse health effects observed in epidemiological studies. The inflammatory potential of particles has been linked to chronic pulmonary diseases, but has also been suggested to contribute to atherosclerosis and acute cardiac effects [20,22,23]. Particle-induced cytotoxicity may be involved in tissue damage in the lung and in other organs, whereas the carcinogenic risk primarily is linked to genotoxiciy [17,24]. Markers of negative health effects (i.e. oxidative stress, inflammation, cytotoxicity and genotoxicity) are commonly monitored in cultured cells (*in vitro*), acute and chronic animal models (*in vivo*) or voluntary individuals in exposure chambers (*in vivo*) to study the effects of particles on human health.

The two previous wood smoke reviews focused on the health effects of residential wood smoke particles based on epidemiological studies [4,5] and experimental studies [5], whereas the present review focuses on the physicochemical properties of the particles, but from a health based perspective. Naeher et al. (2007) concluded that wood smoke may affect pulmonary immune defence mechanisms, with the lung macrophages as a likely target for wood smoke induced immunotoxicity, based on *in vivo *toxicological studies of wood smoke [5]. However, the combustion conditions used to generate wood smoke particles and their physicochemical properties were not discussed, neither was the relevance of these particles with respect to ambient exposure. In the end of their paper Naeher et al. (2007) recommended topics for further research, including; i) 'Better understanding of the similarities and differences of smokes generated by combustion of different categories of biomass in different conditions (...)' and ii) 'Source and exposure apportionment studies to determine the degree to which residential wood combustion contributes to both indoor and outdoor particle exposures (...)'. Although further research is necessary, a notable amount of information is available in the literature concerning both topics. In the present review, we summarise current knowledge on physicochemical properties of PM from residential wood combustion in developed countries with focus on how these properties change with varying combustion conditions and their relevance to human exposure. We also discuss the combustion conditions and the resulting particle properties applied in recent experimental studies of the biological effects of wood smoke, and the relative toxicity of different types of wood smoke particles. The review is organized according to the following outline:

Particle characteristics relevant for health effects

Brief introduction to how the physicochemical properties of particles may influence their biological effects

Physical and chemical characteristics of wood smoke particles

Summary of the current knowledge on the physicochemical properties of wood smoke particles from different combustion conditions, organised into three different particle classes:

- spherical organic carbon particles

- soot particles/carbon aggregates

- inorganic ash particles

Wood smoke exposure

The exposure studies are reviewed to investigate to what extent they provide information about the physicochemical properties of the wood smoke particles

Emissions from different wood combustion appliances

As an alternative to the exposure studies, the emission factors, activity data and emission characteristics of different types of wood combustion appliances are combined to obtain information about the type of wood smoke particles we are exposed to

Transformation of wood smoke emissions in the atmosphere

Discussion of the influence of atmospheric transformations on the physicochemical properties of wood smoke particles and its potential influence on their biological effects

Experimental studies of wood smoke toxicity

Discussion of the combustion conditions and the resulting particle properties applied in recent experimental studies, divided into three parts:

- human inhalation studies

- *in vivo *animal studies

- *in vitro *studies

Summary and conclusions

## Particle characteristics relevant for health effects

The adverse health effects of inhaled particles are highly dependent on the deposition and retention of particles in the lung. The deposition probability and deposition site of particles is governed by their aerodynamic properties, such as size, density and shape, but also by other physicochemical properties such as hygroscopicity (i.e. water uptake) [25,26]. Experimental studies have identified a range of physicochemical properties that influence the toxic and inflammatory potential of PM, and possibly particle-induced health effects (reviewed in [16,17,27,28]). Since these data are discussed in detail in several reviews, they are only described in brief in the following. The most relevant particle properties and a selection of references are summarized in Table 1.

**Table 1:** Physicochemical properties reported to influence the biological effects of PM in experimental studies

**Physicochemical properties**	**References**
Particle size	[29-32]
Surface area per mass	[32-34]
Crystal structure	[35-39]
Chemical composition	
- metals	[41,42]
- organic compounds	[40,43-45]
Solubility	[50,51]

The table lists the most relevant physicochemical particle properties and the references used in the text. For a more comprehensive reference list please refer to one of the reviews [16,17,27,28].

Small particles, exhibiting a large surface area per mass, have been found to induce a more pronounced pro-inflammatory response than larger particles of the same material. This has been demonstrated in both *in vitro *and *in vivo *experiments where ultrafine particles are more potent in inducing inflammatory responses than fine particles [29-32]. Consequently, surface area has been suggested as a new dose metric for the inflammatory effects induced by low-solubility low-toxicity particles *in vitro *and *in vivo *[32-34]. However, particle structure, surface properties and chemistry may override the importance of particle size and surface area. For example, inflammation and cytotoxicity after exposure to ultrafine TiO_2 _has been found to depend on crystal structure (anatase vs. rutile) rather than size and surface area [35,36]. Furthermore, the inflammatory, cytotoxic and genotoxic responses to quartz particles were reduced by surface coating, indicating that surface properties were important for the toxicity of quartz [37-39]. With respect to chemical composition, the content of metals such as vanadium, zinc, iron, copper and nickel, as well as the content of organic compounds such as polycyclic aromatic hydrocarbons (PAHs), seem to influence the particle-elicited health effects [17,40-42]. Quinones, a special group of carbonyl containing PAH compounds, have recently been pointed out as particularly reactive organic components of PM with potential to produce reactive oxygen species (ROS) and to induce oxidative stress via their redox capacity [43]. Accordingly, various oxy-PAHs, including quinones, were found to be involved in inducing cellular oxidative stress in a murine monocyte-macrophage cell line during exposure to organic extracts of wood smoke and diesel exhaust particles [44,45]. However, the organic fraction of particles from various sources comprises a large number of compounds besides PAHs, such as aldehydes, ketones, organic acids and various chlorinated organics [5,46,47], and the biological effects of many of these compounds, and their contributions to particle-induced inflammation, are largely unknown.

Solubility is another property that may influence the toxicity of PM. For particles that dissolve upon contact with aqueous solutions, such as most salt particles, cellular uptake of dissolved ions may occur through ion channels. In contrast, insoluble particles are usually taken up by phagocytosis, which subsequently may initiate a cascade of intracellular signalling [48]. Organic compounds, on the other hand, can enter cells directly through the cell membrane by a partitioning process [49], which in turn may result in activation of other intracellular signalling pathways. Insoluble particles exert a prolonged exposure, while dissolved particulate material is likely to be cleared more rapidly. *In vitro *studies indicate that insoluble nickel compounds are more cytotoxic than soluble nickel salts [50]. On the other hand, the *in vitro *cytotoxicity of manufactured nanoparticles was greater for partly soluble than insoluble particles [51]. Thus, for different types of particles the solubility seems to influence the particle-induced cytotoxicity to different extents.

## Physical and chemical characteristics of wood smoke particles

**Figure 1:**
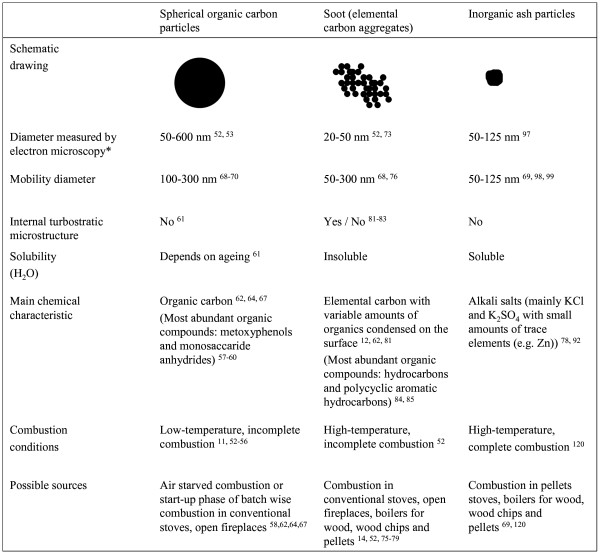
**The physicochemical characteristics of the three classes of wood combustion particles**. The numbers refer to the references used in the text. * For the aggregated soot particles the listed diameter refers to the primary particle diameter.

The physical and chemical properties of wood smoke particles emitted during various combustion conditions differ considerably. Fine particles (equivalent aerodynamic diameter < 2.5 μm, PM_2.5_) emitted from residential wood combustion appliances may be divided into three typical classes based on chemical composition and morphology; spherical organic carbon particles, aggregated soot particles and inorganic ash particles. The physicochemical properties of these three classes are described in the following sections, and summarised in Figure 1. It should be pointed out that in real combustion situations, especially during transient cycles, the particle classes may co-exist and interact. Since the combustion conditions in an appliance change during a burn cycle, especially during batch-wise combustion of wood logs, the emissions are likely to contain several of the defined particle classes.

### Spherical organic carbon particles

Burning wood of poor quality (e.g. high moisture content), overloading the firebox or insufficient air supply, are examples of conditions that can lead to incomplete combustion, characterised by low temperature [11]. In a conventional wood stove without modern combustion technology, emissions from such poor combustion conditions (low temperature, air deficiency and/or poor mixing) are dominated by spherical organic carbon particles with diameters that have been measured to be between 50 and 600 nm by electron microscopy [52,53]. Spherical organic carbon particles have also been observed during smouldering combustion [54-56], and are therefore likely to be emitted from open fireplaces. The origin of this organic material is the thermal degradation products of the wood constituents (i.e. cellulose, hemi-cellulose and lignin) that are released at low temperatures (300-500°C) without being further combusted due to poor mixing conditions.

Freshly generated particles from smouldering combustion contain large amounts of highly oxygenated water-soluble organic species, including monosaccharide anhydrides and methoxyphenols [57-60]. During ageing in the atmosphere (> 10 min) insoluble 'tar-balls' may be formed through polymerisation of primary emitted organic matter [61]. These tar-balls contain low levels of elemental carbon and lack the internal turbostratic microstructure exhibited by the primary particles of carbon aggregates generated at higher temperatures [61]. Particles from incomplete combustion are also characterised by a low content of inorganic constituents such as potassium, sulphur and chlorine [12,62,63]. Wood smoke emissions contain a large number of organic compounds, and detailed chemical speciation of several hundred individual compounds has been reported [57,58,64]. Using on-line aerosol mass spectrometry (AMS), Weimer et al. (2007) showed that organic emissions, particularly those with signatures similar to levoglucosan, were strongly enhanced during the start up phase. The mass spectra recorded during the smouldering phase were, in contrast, dominated by highly oxygenated species [65]. However, the changes in organic chemistry for different combustion conditions and temperatures and for the various phases of the combustion cycle are not well described in the literature.

The carbon present in combustion particles can be classified as either organic or elemental carbon, and may be determined in thermal/optical carbon analysers. Organic carbon (OC) comprises hundreds to thousands of organic compounds, whereas elemental carbon (EC) is defined as the carbon that is not organic, but EC can also be characterised as refractory carbon [66]. The sum of the organic and elemental carbon in a sample is defined as the total carbon (TC). For low-temperature combustion in conventional stoves, the reported ratios of elemental to total carbon (EC/TC) range from 0.01 to 0.11 [62,64,67], confirming that PM from these combustion conditions are dominated by organic carbon. It should be kept in mind though that different measurement techniques give rise to large differences in the EC/TC ratio [66], hence great caution should be taken when comparing such data.

The mobility equivalent diameter, which determines the deposition by diffusion in the human lung (typically important for mobility diameters below about 500 nm), equals the physical diameter for spherical particles. The count mean diameter (CMD) of particles from low temperature biomass combustion has been found to range from 100-175 nm [68,69]. Similarly, Hueglin et al. (1997) measured mobility sizes with CMD between 200 and 300 nm during the start up phase of a residential wood stove, when organic emissions are expected to dominate [70]. Thus, the CMD seems to range from 100 to 300 nm for spherical organic carbon particles. The aerodynamic equivalent diameter determines particle deposition by sedimentation in the lung (typically important for aerodynamic diameters larger than about 200 nm). Since these spherical organic carbon particles have densities of around 1-1.5 g/cm^3 ^[71], the CMD based on aerodynamic equivalent diameter is slightly larger than the CMD based on mobility diameter.

The organic compounds from wood combustion are not only emitted in the particulate phase, but also in the gas phase. In the hot flue gas leaving the combustion chamber of boilers and stoves, most of the organic material is present in the gas phase, but can condense on existing particles (e.g. soot and/or inorganics) during cooling in the heat exchanger and chimney [13,57,64]. Atmospheric processes, for example reactions with OH and O_3_, can result in reaction products with lower vapour pressure that may condense onto existing particles through formation of secondary organic aerosols [72]. There is still insufficient knowledge about the relative contributions of primary emissions and secondary particle formation to the total particulate organic carbon from biomass combustion. It should be pointed out that the gas to particle partitioning of organic compounds depends relatively strongly on concentration. To accurately represent the particle phase of primary organic aerosols from biomass combustion, measurements should preferentially be made at conditions relevant for ambient air.

### Soot (Elemental carbon aggregates)

During incomplete combustion with air-starved conditions at higher temperatures (~800-1000°C), PM emissions are more dominated by solid carbon aggregates (soot). These consist of a large number of primary spherical carbon particles with diameters that have been measured to be between 20 and 50 nm by electron microscopy [52,73]. The formation of soot is very complex and Bockhorn has given a well adapted soot formation pathway, via polycyclic aromatic clusters, particle inception, surface growth and coagulation [74]. Carbon aggregates of soot may be emitted during incomplete combustion in conventional wood stoves and masonry heaters [52,75,76], from open fireplaces [14] or during incomplete combustion in boilers for wood, wood chips or pellets [77-79].

In general, soot can contain some percent of hydrogen, originating from the primary aromatic compounds, and is subsequently more or less graphitized in the combustion process. Primary particles of soot have been reported to exhibit an internal turbostratic microstructure, consisting of a concentric arrangement of layer planes with a two dimensional graphitic structure, lacking the ordered stacking of graphite, and thus its three dimensional structure [80]. Kocbach et al. (2006) observed a turbostratic microstructure consisting of concentric carbon layers surrounding a single nucleus in primary particles from incomplete high-temperature wood combustion by high resolution transmission electron microscopy (HR-TEM) [81]. In the same study, the graphitic character, defined as the degree of similarity to the structure of graphitic carbon, was investigated by selected area electron diffraction (SAED) and electron energy loss spectroscopy (EELS). Wood smoke particles from high-temperature combustion were found to have graphitic character similar to that of traffic-derived particles, confirming the observations by HR-TEM. In contrast, Braun and colleagues recently reported that particles from a range of residential wood stoves did not have a graphitic character or a less graphitic character than diesel exhaust particles by application of near-edge X-ray absorption fine structure spectroscopy (NEXAFS) [82,83]. Whereas the wood smoke particles in Kocbach et al. (2006) were collected by aerosol sampling, Braun and co-authors analysed samples collected either from the interior walls of various wood stoves or from chimneys. The differences in applied collection methods could lead to a selection of different populations of particles. This might explain the conflicting results concerning the graphitic structure of wood smoke soot particles. The present data is insufficient to conclude on a possible difference in the graphitic character of soot particles from different combustion conditions.

Aggregated soot particles contain higher levels of elemental carbon and lower levels of organic carbon compared to carbonaceous particles emitted at lower temperatures, and the EC/TC ratios for incomplete high-temperature combustion in conventional stoves and masonry heaters have been reported to range from approximately 0.5 to 0.75 [12,62,81]. Both the concentration and the relative contribution of various particle associated organic compounds change with combustion temperature. Overall, the total concentration of non-combusted organic matter in the emissions decreases with increasing combustion temperatures, and the primary organic pyrolysis products formed at lower temperatures are "transformed" to purer aromatic hydrocarbons at higher temperatures. Accordingly, the content of methoxyphenols decreases with increasing combustion temperature, whereas the levels of PAHs increase [84,85]. Thus, soot emitted from different combustion conditions may differ in organic chemistry. The most abundant PAHs in wood smoke emissions are naphthalene, acenaphthene, fluorene, phenanthrene, anthracene, fluoranthene and pyrene [15,47,64], but with regard to carcinogenicity, benzo(a)pyrene (B(a)P) and fluoranthene seem to be the most important compounds in wood smoke emissions [47,86,87]. Although a rather extensive amount of work has been performed to characterise the organic fraction of wood smoke [47,57,58,64,88], little information is so far available concerning compounds that influence the biological effects of wood smoke particles and on how the organic composition varies with the combustion temperature. However, fractionation of organic extracts, chemical analyses and measurements of oxidative stress were recently combined in order to identify the organic compounds involved in the biological effects of wood smoke particles [44]. In that study, oxy-PAHs and quinones were found to contribute to oxidative stress. Interestingly, emissions of oxy-PAHs have been reported to increase with increasing wood combustion temperature [85].

A particle diameter is hard to define for aggregated particles such as soot, and the mobility equivalent aggregate diameter for soot from wood combustion has been found to vary considerably between different studies; from 50 to 300 nm [68,76]. The aerodynamic equivalent diameter of soot from wood smoke has not been reported in the literature, but may be considerably smaller than the mobility equivalent diameter [89]. Condensation of organic compounds onto soot agglomerates may lead to a transition from highly agglomerated to compact particles. This has been demonstrated for soot from other sources during interaction with water or H_2_SO_4 _[90,91]. The present knowledge of the morphology and the mobility and aerodynamic diameters of aggregated particles from wood combustion is, however, limited.

### Inorganic ash particles

Combustion of pellets, wood chips and wood logs in boilers or stoves with modern technology provides favourable combustion conditions with high temperatures (> 900°C), good oxygen supply and sufficient mixing between combustable gases and air in the combustion chamber. This results in almost complete combustion and the emissions are dominated by inorganic ash particles, such as the alkali salts of potassium/sodium-sulphates, chlorides and carbonates [78,92]. The content of organic and elemental carbon can be below 1% of the particle mass emitted during these favourable combustion conditions [69]. Fine particles emitted during combustion of some types of wood and bark pellets may also contain phosphorous, which is probably related to elevated combustion temperatures [93]. It is also believed that potassium phosphates may be present in fine particles during combustion of more phosphorous rich (non-woody) biomass, as demonstrated during combustion of agricultural fuels in some recent studies [94-96].

Studies using electron microscopy have revealed that the fine inorganic ash particles emitted from complete combustion conditions have a sphere-like shape with diameters between 50 and 125 nm [97]. The corresponding mobility diameters have been measured to be in the same size range [69,98,99]. Since mobility diameters are close to the physical diameter for compact particles, the inorganic ash particles from biomass combustion are also likely to have physical diameters in the same range. Aerodynamic diameters may be calculated assuming an effective density of about 2.0 g/cm^3 ^[68,70]. For example a particle with an equivalent mobility diameter of 100 nm and effective density of 2.0 g/cm^3 ^would have an equivalent aerodynamic diameter of 168 nm. Overall, the particle morphology and size distribution has been relatively well described for inorganic ash particles.

Inorganic ash particles such as potassium sulphates and chlorides have rather high hygroscopic growth factors and are mainly water soluble. This solubility may affect the biological effects induced by these particles in two manners; (i) hygroscopic particles may grow at the high humidity in the respiratory tract, which can reduce the deposition probability and may alter the deposition site [25,100] and (ii) the solubility may increase the clearance rate from the lung. In addition, the solubility of PM may affect the biological effects on a cellular level, for instance with respect to uptake mechanisms and activation of intracellular pathways.

In addition to the three classes of particles described above, coarse inorganic fly-ash particles with diameters larger than 1 μm, containing refractory species such as calcium, magnesium, silicon, phosphorus and aluminium, have been detected in emissions from large scale grate fired biomass boilers [78,101] and wood chip burners [70]. In grate fired appliances, the air is supplied to the combustion chamber through a grate beneath the chamber. The coarse fly-ash particles are entrained from the fuel bed and their emissions may therefore be strongly dependent on the primary air flow through the grate [98].

## Wood smoke exposure

In order to evaluate the negative health effects that may be associated with exposure to wood smoke particles, it is necessary to determine the human exposure to these particles. The number of studies regarding ambient wood smoke exposure in developed countries increases rapidly. Source apportionment studies have estimated that wood/biomass combustion contribute with 10-40% to the fine particle concentrations (PM_2.5_) in large cities such as Seattle, Phoenix, Beijing, Prague and Helsinki [102-105]. Residential wood combustion has also been reported to contribute substantially to increased levels of air pollution locally, both with respect to increased levels of PM_2.5_, the organic particle fraction, particle bound PAH and volatile organic compounds [106-111]. The contribution of wood smoke to ambient air pollution is, however, highly dependent on season, time point and week day [105,112].

In general, people in developed countries spend the majority of their time indoors. For instance, the participants in a recent Swedish study reported that they spent more than 90% of their time indoors and around 60% at home [113]. Thus, the indoor particle levels have a large impact on human exposure. The penetration of wood smoke from ambient sources to indoor environments has not been investigated in any detail. However, both the personal exposure and the indoor concentrations of particle associated K, Ca, Zn, and possibly Cl, Mn, Cu, Rb, Pb and black smoke (~soot), were found to be increased in homes heated with a wood stove or boiler [114]. Personal exposure and indoor levels showed high correlations for all elements, and the personal exposure levels were usually higher than or equal to the indoor levels, but the associations between personal exposure and outdoor levels were generally weak [114]. Residential wood combustion also increased personal exposure to 1,3-butadiene as well as indoor levels of 1,3-butadiene and benzene and possibly acetaldehyde [115]. The cancer risk from these compounds due to wood smoke exposure in developed countries was estimated to be low [115]. In the same study, the levels of B(a)P and several other PAHs were found to be significantly higher (3- to 5-fold) in homes with wood combustion appliances compared to homes without [87]. While phenanthrene made the largest contribution to the total PAH concentration in indoor and outdoor air, most of the cancer potency was due to B(a)P (about 60%) and fluoranthene (about 20%). Moreover, the median indoor B(a)P concentration in the homes with wood combustion appliances (0.52 ng/m^3^) was 5 times higher than the Swedish health-based guideline of 0.1 ng/m^3^.

The physicochemical characteristics of ambient wood smoke particles are highly dependent on factors that vary between locations, such as the relative numbers of different types of residential combustion appliances, and on factors that vary with both time and location, such as the combustion activity (e.g. use frequency and burn rate), the wood species and wood quality. The contribution from residential wood combustion to ambient, indoor and personal wood smoke exposure is commonly estimated by application of various markers for wood smoke, such as the content of organic and elemental carbon, specific organic compounds (levoglucosan, 1,3-butadiene, benzene, or PAHs) or metals (K, Ca, Zn) [87,105,114-117]. However, these markers provide limited information regarding the exposure to the different classes of wood smoke particles, as they are usually only representative for one of the three classes of residential wood combustion particles. Thus, a broader range of wood smoke markers with specificity for each of the three classes of wood smoke particles should be applied in future exposure studies. This would provide a better characterisation of wood smoke exposure in epidemiological studies, and also a better basis for choosing relevant particles in experimental/toxicological studies. Further characterisation of the personal and indoor wood smoke exposure is also necessary, since we generally spend more than 60% of our time indoors at home.

## Emissions from different wood combustion appliances

**Figure 2:**
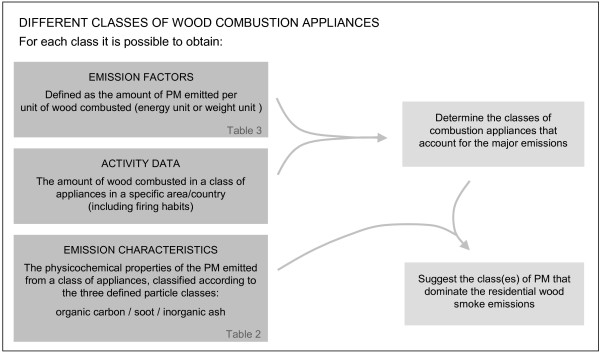
**Flowchart illustrating how information about the physicochemical properties of ambient wood smoke particles may be obtained from data collected for individual classes of combustion appliances**. See text for explanation.

The physicochemical properties of ambient wood smoke particles depend on the wood smoke emissions to ambient air. Data collected for individual classes of combustion appliances may be applied to obtain information about the physicochemical characteristics of residential wood smoke emissions in a specific area, as illustrated in the flowchart in Figure 2. For each class of wood smoke appliances it is possible to determine emission factors, activity data and emission characteristics. By combining the activity data with the emission factors the classes of combustion appliances that account for the majority of the emissions are determined, and in combination with the emission characteristics the classes of PM that dominate the residential wood smoke emissions can be suggested. This approach provides a rough estimate for the main characteristics i.e. the particle class(es) dominating the emissions, but application of exposure studies provide more relevant chemical characterisation and also includes the atmospheric modifications that have occurred in the time span between emission and exposure.

### Emission characteristics

In this section, the class of PM (i.e. organic carbon/soot/inorganic ash) that dominates the emissions from the different types of combustion appliances is suggested based on the available data for EC/TC ratios and morphology of the emitted PM (Table 2). If the data is limited a suggestion is made based on knowledge about the combustion conditions in that type of appliances.

**Table 2:** Emission characteristics for the different classes of wood combustion appliances

**Type of combustion appliance**	**Particle class(es) dominating the emissions**	**References**
Open fireplaces	organic carbon/soot	[14,58,62,64,67]
Conventional wood stoves	organic carbon/soot	[12,62,64,67,81]
Masonry heaters	organic carbon/soot	[11,76,119]
Conventional boilers for wood logs	organic carbon/soot *	
Modern wood stoves	inorganic ash/organic carbon/soot *	
Modern boilers for wood logs	inorganic ash/organic carbon/soot *	
Pellet stoves and boilers	inorganic ash	[69,120]

Based on the available data on the physicochemical properties of particles emitted from different types of combustion appliances we have suggested the class(es) of particles that are likely to dominate the emissions. The references used to support the text are included in the table.

* Limited data available, see text for details

#### Open fireplaces

Wood combustion in open fireplaces is a mixture of flaming and smouldering combustion. These emissions are therefore likely to be dominated by spherical organic carbon particles and soot. Scanning electron microscopy of samples from a range of fireplace emissions suggested that carbon aggregates (soot) was the dominating particle class [14]. However, the reported EC/TC ratios, ranging from 0.04 - 0.46 [14,58,62,64,67], indicate that organic carbon is the major component of PM emissions from open fireplaces. A possible explanation for the discrepancy between the EC/TC ratios and the morphology observed by electron microscopy may be condensation of organic carbon onto soot particles. Overall, the reported data on emission characteristics from open fireplaces suggest that the contribution to ambient air from this class of wood combustion appliances is a mixture of soot and organic carbon.

#### Conventional wood stoves

As discussed previously, particle emissions from incomplete low-temperature combustion conditions are dominated by spherical organic carbon particles and low levels of elemental carbon (EC/TC ratios 0.01-0.11), while soot and high EC/TC ratios (0.50-0.75) characterise emissions from incomplete combustion at higher temperatures [12,62,64,67,81]. Kocbach et al. (2005) observed soot, but not spherical organic carbon particles, in ambient samples collected in two areas dominated by smoke from conventional stoves. The samples comprised emissions from different combustion conditions and several wood species, suggesting that the contribution from conventional stoves to ambient air was mainly soot [52]. In contrast, another study indicated that spherical carbon particles observed in ambient air samples originated from household wood combustion [61]. Although soot seems to constitute a large part of the emissions from conventional wood stoves, organic carbon, either condensed onto soot or as individual spherical carbon particles, also appears to be an important contributor to the particle emissions from this class of combustion appliances. Gaseous organics emitted during poor combustion conditions are also likely to contribute to the particulate OC levels due to formation of secondary organic aerosols [118].

#### Conventional wood log boilers and masonry heaters

Conventional wood log boilers and masonry heaters can be defined as appliances without new technology, such as down-draft combustion, sucking fan and electric combustion control. Conventional wood log boilers may be installed with a water heat accumulation tank, which improves the user comfort and combustion efficiency considerably. In Sweden, less than 30% of the households with wood log boilers have a water heat accumulation tank. Analyses of carbon content or morphology of the particulate emissions from wood log boilers have not been reported in the literature. However, since the combustion conditions (e.g. temperature, residence time and mixing) in such systems can vary significantly, the emissions can be expected to vary with respect to the fractions of organic carbon, elemental carbon (soot) and inorganic ash constituents. In general, the PM is dominated by carbonaceous material and for conventional masonry heaters, the EC/TC ratios have been reported to range from approximately 0.10 to 0.35 in both field and laboratory studies [11,76,119]. Thus, the emissions from conventional wood log boilers and masonry heaters are likely to be dominated by soot and organic carbon.

#### Modern stoves, masonry heaters and boilers for wood logs

In "modern" residential combustion appliances for wood logs, the applied combustion technology leads to improved combustion conditions with good burn out and low emissions of PM [120]. The emissions from modern appliances for combustion of wood logs are dominated by inorganic ash during ideal operation, and organic carbon and soot may constitute less than 10% of the emitted particle mass [120]. However, during the start-up phase and during low burn-rates, the combustion performance can be deteriorated causing increased emissions of both organics and soot. Moreover, the emissions from modern appliances for wood logs may increase ten-fold if they are not operated appropriately [118] and then the emissions are most likely dominated by soot and organic carbon rather than inorganic ash. The data reported concerning detailed chemical composition of the PM for modern wood boilers and stoves are still very scarce.

#### Pellets stoves and boilers

Wood pellet boilers and stoves can in general be considered as "modern" technology with high combustion efficiency, and situations with poor combustion conditions are assumed to be very rare in these systems due to for instance the homogeneous character of the fuel, continuous fuel feeding and fan driven air supply [118]. Based on laboratory studies, the emitted PM from these appliances is therefore assumed to be dominated by inorganic ash and to contain very low levels of elemental and organic carbon (TC ~5 - 12% and EC/TC ~0.65 - 0.80) [120]. The total carbon level may be as low as < 1% [69]. However, the efficiency of these appliances may be deteriorated if they are installed or operated in an inappropriate manner, this could possibly cause emission of soot or organic carbon.

Overall, the emissions of gases and PM from the various types of combustion appliances have been rather well described, although the data available for modern wood stoves and boilers for wood logs have some limitations. A more detailed characterization of the variations in particle properties between the different types of appliances is however still missing. Efforts should be made to resolve this issue, since a more complete characterisation of the variation in particle properties would provide a better basis for an evaluation of the impact of wood smoke exposure on human health.

### Emission factors and estimates

The emission factors of PM for different classes of residential biomass combustion appliances have recently been discussed in several reports [118,120-122], and are summarised in Table 3. Conventional stoves and boilers for wood logs account for the highest emission factors, followed by open fireplaces and modern stoves and boilers and finally pellet stoves and boilers. Generally, the ranges of emission factors reported for the various classes of appliances are very large. This variation is partly due to application of different measuring techniques; both sampling of particles in the chimney at gas temperatures of 120-160°C and sampling of particles in a dilution tunnel at lower temperatures (< 35°C) are commonly applied. Application of a dilution tunnel allows for condensation of organic compounds onto the particles, and the resulting emission factors can be up to 10 times higher than the factors based on collection of particles in the undiluted chimney gas [118,120]. However, if even higher dilution ratios are applied (above 20:1) the emission estimates for organic carbon may decrease with increasing dilution ratios [123]. The fraction of primary formed organics (i.e. products of incomplete combustion) which partitions to the particle phase is strongly dependent on both concentration and temperature [118,123-125]. Thus, to accurately quantify the primary organic particle phase fraction in atmospheric wood smoke pollution, dilution conditions close to ambient should be applied. It should also be kept in mind that wood combustion appliances are often pre-heated when their emission factors are determined. This is in contrast to real-life wood combustion, where the burning of wood starts in a cold stove. Since organic compounds are likely to dominate the emissions from a cold stove, this procedure may contribute to an underestimation of the real-life emissions of organic carbon.

**Table 3:** Emission factors for different types of residential combustion appliances

**Type of combustion appliance**	**Reported emission factors**	
	
	**Approximate range** **(mg/MJ)**	**Reported data** **(mg/MJ)**
Open fireplaces	160 - 910	800 ^a^
		160 - 447 ^b,1^
		860 - 910 ^b,2^

Conventional wood stoves	50 - 2100	700 ^a^
		94 - 650 ^b,1^
		50 - 1932 ^b,2^
		100 ^c^
		150 - 2100 ^d^

Other conventional stoves, including masonry heaters and sauna stoves	30 - 140	140 ^a^
		30 - 100 ^c^

Conventional boilers for wood logs		
*without accumulator tank*	50 - 2000	700 ^a^
		300 - 2000 ^b,1 and 2^
		1300 ^c^
		300-900 ^d^
*with accumulator tank*	50 - 250	80 ^a^
		50 - 300 ^b,1 and 2^
		95 ^d^

Modern wood stoves	34 - 330	34 ^c^
		330 ^d^

Modern boilers for wood chips or logs	5 - 450	5-450^b,1^
		20 - 25 ^c^
		30-100 ^d^

Pellet stoves and boilers	10 - 50	30 ^a^
		10 - 50 ^b,1 and 2^
		20 ^c^
		30 ^d^

Emission factors are reported as mg particles emitted per MJ of fuel burnt (MJ = Mega Joule)

^a ^mean emission factors based on available literature, as reported in [121].

^b ^range of emission factors based on data from members of the International Energy Agency, as reported in [118]. 1 = measurement of particles at temperatures > 100°C, 2 = measurement of particles in dilution tunnel at temperatures < 100°C.

^c ^range of emission factors [120].

^d ^data from [122].

The operation conditions, e.g. ideal, typical or poor operation, also have great impact on the measured emission factors for appliances fired with wood logs, and the emission factors for wood stoves and boilers have been estimated to increase with a factor of 10 during typical operation as compared to ideal operation [118]. In addition, the large variations in combustion technology within each class of combustion appliances also contribute to increased variation in the reported emission factors. The data in Table 3 suggest that the emission factors for residential wood combustion appliances are highly uncertain, and their uncertainty was recently estimated to be ± 54 - 88% for Finland (95% confidence interval) [121].  As illustrated in Figure 2, emission estimates for the different class of combustion appliances may be obtained by combining the activity data with the corresponding emission factors. In comparison to the large uncertainty determined for the emission factors, the uncertainty related to the activity in the domestic wood combustion sector was found to be considerably lower (± 10%), while the uncertainty regarding the activity in different types of combustion appliances were between ± 15% and ± 25% [121].

Recently, the number of biomass combustion appliances, the activity, and the calculated estimated emissions based on emission factors were summarized for several European countries (Denmark, Finland, Norway, Sweden, Germany and Switzerland) [120-122]. To our knowledge, emission estimates for the different classes of wood combustion appliances have not been calculated for other European countries, the US, Canada, Australia or New Zealand. As mentioned above, considerable uncertainty is associated with these numbers, they do however provide an estimate of the residential wood combustion emissions. All studies reported that wood logs is the most commonly applied fuel in biomass-based residential heating, and that the majority of the wood was combusted in conventional stoves and manually fed boilers [120-122]. Since these combustion appliances have high emission factors (Table 3), they also account for the majority of the emissions of PM from residential biomass combustion, generally more than 80% [120-122]. Due to low activity or low emission factors, fireplaces and modern wood log appliances account for less than 15% of the residential biomass emissions in the Nordic countries [121,122]. This is in contrast to the US, where open fireplaces are considered to be one of the major contributors to residential wood smoke emissions [12].

Over the last 10-20 years, the development of new combustion technologies for densified wood fuels, such as pellets, has been considerable in several countries, like Sweden, Austria and Germany [10]. Although log wood is still the dominating fuel type in most European countries, wood pellets have gained increasing relevance and this trend is expected to continue [120]. Due to their low emission factors and the relatively low number of appliances, the relative contribution from pellets burning to the total biomass combustion emissions is generally below 10% [120-122]. The share of modern biomass combustion appliances, for both wood log and pellets, is likely to grow steadily, particularly due to replacement of older stoves/boilers and due to conversion from oil and electricity. The relative contribution from these appliances to the total residential wood smoke emissions is, however, likely to remain low due to their low emission factors.

### Emissions of the different classes of wood smoke particles

As discussed in the previous section, conventional wood stoves and boilers for wood logs account for the majority of the domestic biomass emissions to ambient air in Europe. Since these emissions consist of variable fractions of soot and organic carbon depending on combustion appliances, operation and fuel quality, these two classes of particles are likely to dominate the emissions in European countries. The organic compounds may be condensed onto soot and/or inorganic particles or be present as individual spherical organic particles in emissions from very poor combustion conditions.

With respect to relevance for experimental studies, particles generated solely during smouldering combustion, not containing soot, seem to be more representative for bush and structural fires, and hence for fire fighter exposure, than for residential wood smoke exposure. In addition, smouldering combustion and spherical organic carbon particles are also relevant for the domestic exposure in developing countries, since open fires that provide poor combustion conditions are commonly burned indoors in these countries. Inorganic ash particles are primarily emitted from pellets stoves and boilers and from modern wood log boilers under optimal firing. Due to the low emission factors of these appliances, inorganic ash particles make a small contribution to ambient wood smoke concentrations presently, but their contribution may increase in the future.

Combining the activity data with the emission factors and emission characteristics for the different types of combustion appliances provides some information about the classes of PM that dominate the residential wood smoke emissions in specific areas/countries. A major limitation of this approach is the high uncertainty associated with the acquired information. In addition, activity data are unavailable for many regions and the emission characteristics are insufficient for some types of appliances. This approach does, however, have promising aspects as it has the potential to provide information about the general type of emissions to a specific area/country without performing time consuming and expensive field measurements.

## Transformation of wood smoke emissions in the atmosphere

The physiochemical properties of ambient particles may change through interaction with atmospheric photo-oxidants (e.g. OH, O_3_, NO_3_. NO_2_), acids (e.g. HNO_3_, H_2_SO_4_), water and UV radiation [126]. Possible atmospheric transformations include altered size, morphology and chemical composition [90,91,127-129]. Few studies have investigated the atmospheric transformations of wood smoke particles, but the metoxyphenols present in wood smoke particles have been suggested to enhance the photochemical degradation of PAHs [128]. In addition, more volatile compounds have been reported to condense onto particles, and heavy compounds to be photo-degraded into lighter ones [129,130]. Photo-oxidation of wood stove emissions at atmospherically relevant or slightly elevated concentrations in a Teflon chamber has been found to increase the organic aerosol mass by a factor of 1.5-2.8 [130]. The condensed material was highly oxidised, distinctly different from the primary organic particle mass. Less than 20% of the formed secondary aerosol mass could be explained by known pre-cursors, indicating involvement of large classes of organic compounds [130]. These effects are qualitatively similar to those previously reported for diesel exhaust [125]. It is obvious that more research is needed on this topic, for example on how the combustion conditions influence the formation of secondary organic aerosols [131].

Atmospheric alterations could affect the biological activity of PM, and cause either increased or decreased potency with respect to mutagenicity, inflammatory potential or toxicity [132-136]. The performed studies also suggest that the effect of ageing on the biological activity could be related to the particle source.

The present data on atmospheric alterations of particulate matter suggest that it is necessary to take into account the atmospheric alterations of the emitted particles in order to elucidate the potential health effects of wood smoke. Further studies are necessary both with respect to changes in physicochemical particle properties but also with respect to the influence of these changes on the biological effects.

## Experimental studies of wood smoke toxicity

The physicochemical properties of wood smoke particles applied in experimental studies, have not been discussed in recent reviews of health effects of wood smoke [4,5,137]. As discussed previously, particles generated under varying combustion conditions differ with respect to physicochemical properties, and this may influence their potential to induce biological effects. Therefore, the applied combustion conditions and, if possible, the physicochemical properties of the wood smoke used in recent experimental studies are discussed in this section, and summarised in Table 4.

**Table 4:** Experimental studies of wood smoke toxicity

**Stove/combustion conditions**	**Dominating particle class**	**Model system**	**Biological response**	**Comparison of combustion conditions**	**References**
**Human inhalation studies**					

Conventional wood stove	organic carbon/soot	inhalation, *human*	- inflammation in distal airways - systemic inflammation - blood coagulation - lipid peroxidation - increased oxidative stress ?	-	[7,8,138,139]
Pellets burner/*incomplete combustion*	organic carbon/soot	inhalation, *human*	- increased oxidative stress ?	-	[120,140]

***In vivo *animal studies**					

Conventional wood stove/*mixed burn-cycle*	organic carbon/soot	inhalation, *rat*	- mild chronic inflammation	-	[75]
Conventional wood stove/*incomplete combustion*	organic carbon	inhalation, *mouse/rat*	- allergic airway inflammation - decreased lung function - mild lung inflammation - systemic immunotoxicity - increases in platelet levels	-	[141-145]
Conventional wood stove/*high-temperature incomplete combustion*	soot	footpad immunisation model, *mouse*	- enhanced allergic sensitisation	-	[146]

***In vitro *studies**					

Old boiler, modern boiler, pellets boiler		epithelial cell line, *human*	- genotoxicity - inflammation	no large differences	[147]
Thermolysis of bark/*incomplete combustion*	organic carbon	macrophage-like cell line, *mouse*	- DNA damage - oxidative stress - inflammation	-	[148]
Conventional wood stove/*high-temperature incomplete combustion*	soot	epithelial and monocytic cell lines, *human*	- DNA damage	-	[149]
Modern boiler, conventional wood stove/*normal and poor combustion conditions*	inorganic ash soot, organic carbon	fibroblast cell line, *hamster*	- chromosome breakage - cytotoxicity	organic carbon > soot > ash	[53]
Conventional masonry heater/*normal and poor combustion conditions*		macrophage-like cell line, *mouse*	- cytotoxicity - inflammation: TNF-α MIP-2	poor > normal poor < normal poor > normal	Salonen et al. in [120]
Conventional wood stove/*high-temperature incomplete combustion*	soot	epithelial and monocytic cell lines, *human*	- inflammation	-	[150,151]
Large biomass combustion plant	inorganic ash	epithelial cell line, *human*	- inflammation	-	Bellman el al. in [120]

The table summarizes the studies discussed in the text. Only the endpoints or biological effects that were influenced during exposure to wood smoke particles are listed in the table, not all the endpoints investigated in each study.

### Human inhalation studies

A limited number of human inhalation studies have investigated the negative effects of wood smoke exposure. Barregard and colleagues used a conventional stove to generate wood smoke [7,8,138,139]. The mass concentration (PM_1_) was approximately 250 μg/m^3 ^with levels of B(a)P around 20 ng/m^3 ^and total PAH levels around 800-1000 ng/m^3 ^(sum of 14 measured PAHs). The major inorganic elements, K, Zn and Cl, accounted for less than 6% of the total mass concentration [138], thus soot and organic carbon seemed to dominate the PM inhaled in this study rather than inorganic ash. The geometric mean diameters were 42 and 112 nm in the two different rounds of wood smoke exposure. Blood and urine measurements suggested that wood smoke may be associated with systemic inflammation (the acute phase protein serum Amyloid A and to some extent serum C-reactive protein), blood coagulation (Factor VIII) and lipid peroxidation (urinary excretion of the isoprostane 8-isoPGF2α) [7]. In addition, wood smoke exposure increased markers of inflammatory effects on distal airways (alveolar nitric oxide and Clara cell protein in serum) [8]. Several of these biomarkers are cardiovascular risk factors. The oxidative DNA damage and related repair capacity in peripheral blood mononuclear cells was investigated in the same study. Although wood smoke exposure was followed by significant up-regulation of the repair gene hOGG1, no direct genotoxic effects were observed [139].

Recently, another human inhalation study was performed by a Swedish interdisciplinary group at Umeå University, Umeå University Hospital and Lund University, investigating the effects of wood smoke from an adjusted residential wood pellet burner under low temperature incomplete combustion conditions in a human chamber study [120,140]. The exposures were performed at 224 ± 22 μg PM_1_/m^3 ^where the PM was dominated (~90%) by carbonaceous matter [68]. The preliminary human exposure effect data indicate a moderate response, including increased levels of glutathione which indicates that the antioxidant defense was activated, possibly due to oxidative stress [120,140].

Future human inhalation studies should be designed to compare the effects induced by wood smoke from different combustion conditions, as comparative studies would be a useful tool in the process of targeting strategies for reducing human wood smoke exposure to the appropriate particle fractions.

### *In vivo *animal studies

*In vivo *wood smoke studies in animal models may be divided into exposure conditions relevant for a) fire-fighters or fire victims (studies using high doses and short exposure time) and b) ambient residential wood smoke exposure in developed countries (studies using lower concentrations and acute, intermediate or long-term exposure). The majority of the *in vivo *animal studies using low exposure conditions were performed at the Lovelace Respiratory Research Institute (LRRI, New Mexico, US) [75,141-145]. A conventional wood stove was applied to generate the smoke, using a three-phase burn cycle (kindling, high and low burn rate). Since > 70% of the combustion was performed with a low burn rate, the particles used in these inhalation studies were most likely dominated by spherical organic carbon particles, as supported by the high OC content reported in these studies (90-94% of total carbon content) [143]. However, one early study used particles that were dominated by carbon aggregates (soot) [75]. In light of the discussion in the sections concerning wood smoke exposure, these studies applying particles with very low content of soot (EC/TC ratio < 0.06) may not be fully representative for wood smoke exposure in general in developed countries. Wood smoke-induced effects in mice and rats reported in the studies performed at LLRI include exacerbation of allergic airway inflammation, decreased lung function, mild lung inflammation and toxicity, systemic immunotoxicity and increases in platelet levels [75,141-145]. In contrast to these studies, Samuelsen et al. (2008) used particles from incomplete high-temperature combustion in a conventional wood stove (soot dominated) to investigate the allergy adjuvant effect in mice, and observed enhanced allergic sensitisation after wood smoke exposure [146]. The applied model system, a footpad immunisation model, differed considerably from the model systems used in the studies performed at LLRI, with respect to both exposure route and analysed biological endpoints. This precludes a comparison of the results from these studies.

### *In vitro *studies

The mutagenic potential of wood smoke particles has been relatively well documented in bacterial systems and seems to depend on the PAH content, which is influenced by the combustion conditions [5]. Wood smoke particles have also been reported to induce DNA damage in human monocytic and epithelial cell lines and in a murine macrophage cell line [147-149]. Surprisingly, particles from three different combustion appliances (old boiler, modern boiler and pellets boiler) with varying content of organic carbon showed a similar genotoxic potency [147]. On the contrary, the combustion conditions were found to have great influence on the ability of wood smoke particles to induce chromosome breakage, when investigated by the micronucleus test in a lung fibroblast cell line from Chinese hamsters; particles generated during incomplete combustion conditions induced much higher levels of chromosome breakage than particles generated during more complete combustion conditions [53].

Particles emitted from a variety of stoves and combustion conditions have been reported to increase the release of pro-inflammatory cytokines in different *in vitro *model systems [120,147,148,150,151]. However, only one study compared the influence of the combustion conditions on the inflammatory response. Particles from normal combustion conditions in a conventional masonry heater were found to induce a slightly higher release of the pro-inflammatory cytokine tumour necrosis factor (TNF)-α from a murine macrophage cell line than particles from poor combustion conditions (Salonen et al. in [120]). The latter were, however, more potent inducers of macrophage-inflammatory protein (MIP)-2, the murine analogue of IL-8. One study compared particles emitted from three different combustion appliances, a modern wood pellet boiler, a pellets burner and an old boiler, but the reported differences in inflammatory potential were small [147]. This study only used one concentration and time point in their experiments, which limits the reliability of the presented data, as the relative responses induced by the different wood smoke samples could change with particle concentration and exposure time.

Particles from large biomass combustion plants from combustion of waste wood or bark, consisting mainly of inorganic salts, were found to induce an inflammatory response in a human epithelial cell line, but the same particles did not induce an influx of inflammatory cells to the lungs of rats (Bellmann et al. in [120]). The authors suggested that this may be due to rapid clearance of soluble constituents in the *in vivo *model systems, whereas clearance was not possible *in vitro*.

Particles from incomplete high-temperature combustion were found to induce low cytotoxicity in human monocytic and epithelial cell lines [150,151]. In another study, soot from a poorly operated stove exhibited much higher cytotoxicity than particles from normal combustion conditions in a fibroblast cell line, whereas inorganic particles from complete combustion conditions were even less toxic [53]. Similarly, particles from incomplete combustion conditions induced greater increases in cytotoxicity and programmed cell death (apoptosis) in a murine macrophage cell line than particles from normal combustion conditions (Salonen et al. in [120]).

The organic fraction of wood smoke particles has been suggested to be involved in the release of inflammatory mediators and DNA damage [149-151]. Klippel and Nussbaumer compared the toxicity of the condensable organic matter collected during poor, normal and complete combustion conditions [53]. Interestingly, the condensable organic matter from the three different combustion conditions had a similar toxicity when compared on equal mass concentration, but the amount of condensable organic matter emitted increased with decreasing combustion efficiency. Kubatova et al. (2006) applied a novel method for fractionation of organic extracts in combination with chemical analysis to determine the groups of organic compounds that contribute to cellular oxidative stress. Mid-polarity and non-polar compounds, including oxy-PAHs, were identified as inducers of oxidative stress in a macrophage cell line [44]. Further similar studies are necessary to determine how these groups of compounds or other organic compounds influence a wider range of biological endpoints, but also to determine the influence of varying combustion conditions.

The available literature concerning wood smoke exposure in human volunteers and animal model systems is not sufficient for comparison of the effects induced by particles from different combustion conditions or to discuss the influence of the physicochemical properties on the biological response. However, the number of *in vitro *studies that compare wood smoke particles generated under varying combustion conditions is currently increasing. As discussed above, particles from different combustion conditions seem to induce differential pro-inflammatory response patterns, whereas particles from poor combustion seem to have greater effects on both cytotoxicity and DNA damage than particles from more complete combustion conditions. However, *in vitro *model systems have several limitations. For instance, the particle exposure does not mimic the conditions during *in vivo *exposures and these models also lack the cellular interactions and neurological signals that are of importance in animals. The physicochemical properties of collected particles may also be altered compared to the properties of particles deposited directly from the gas-phase, which occurs during human exposure. In *in vitro *experiments, different particle samples are usually compared on an equal mass basis. However, the pulmonary deposition and retention of particles partly depends on the physicochemical particle properties [25,26]. Thus, during inhalation of equal concentrations of wood smoke particles with different physicochemical properties, pulmonary cells may be exposed to different particle concentrations due to differences in deposition efficiency. This was recently demonstrated for biomass combustion aerosols generated under different combustion conditions [69]. Particles generated during complete combustion conditions (inorganic ash) and particles generated during incomplete combustion conditions (soot/organic carbon) showed relatively low respiratory tract deposition compared to traffic-derived particles due to their size and hygroscopicity [69]. This demonstrates the importance of considering the deposited dose when estimating the toxicological potential of air pollution particles.

In order to target the strategies applied to reduce wood smoke emissions, it is crucial that future toxicological studies provide information about how physicochemical properties, combustion conditions and the type of fuel and combustion appliance influence the toxicity of the emitted particles. We suggest that future toxicological studies perform a minimum of physicochemical characterisation, i.e. determine the fractions of organic carbon, soot and inorganic ash, and perform further characterisation of the organic fraction. We also emphasize the need for further studies comparing wood smoke particles from different combustion conditions generated from the same stove, particularly in *in viv*o model systems.

## Summary and conclusion

### Summary

Wood smoke particles were divided into three classes based on their physicochemical properties; spherical organic carbon particles, soot particles and inorganic ash particles. These particle classes differ with respect to properties that are likely to influence their toxicity, such as size, morphology, internal microstructure, solubility, hygroscopicity, organic chemistry and content of inorganic compounds (Figure 1). Emissions from various appliances often contain several of the defined particle classes.

The reviewed studies of ambient, indoor and personal exposure applied various markers to estimate the wood smoke exposure. These markers are usually only representative for one class of residential wood combustion particles, and therefore provide limited information regarding the physicochemical properties of the particles we are exposed to. However, by considering the physicochemical properties of emissions from different types of combustion appliances (Table 2), and their emission factors (Table 3) and estimates, soot and organic carbon were suggested to be the dominating classes in wood smoke exposure in European countries. Inorganic ash particles, primarily emitted from pellets burners and modern wood log boilers, were found to make a small contribution to ambient concentrations at present, although their contribution is likely to increase in the future.

Only a few experimental studies have compared the biological effects induced by particles from different combustion conditions (Table 4). The conducted *in vitro *studies suggested that the biological potential varied with the combustion conditions; particles from poor combustion induced more severe effects on both cytotoxicity and DNA damage than particles from more complete combustion conditions. However, the current *in vivo *data concerning biological effects of particles from varying combustion conditions is scarce, and further investigations are necessary.

### Conclusion

Epidemiological and experimental studies provide increasing evidence for an association between wood smoke exposure and various health outcomes such as decreased lung function, reduced resistance to infections and increased severity/incidences of acute asthma. Moreover, inhalation studies have demonstrated that wood smoke exposure may induce systemic effects, providing a possible link to cardiovascular effects. The influence of the physicochemical properties of wood smoke particles, and of the combustion conditions, on various biological endpoints is presently largely unknown, although *in vitro *studies suggest that particles from incomplete combustion conditions are more toxic than particles generated under more complete combustion conditions. In order to establish targeted strategies to reduce wood smoke emissions in developed countries, more research is needed concerning the physicochemical properties of the wood smoke particles we are exposed to and the influence of these properties on the induced biological effects. To achieve this, there is need for a stronger collaboration between the different fields of research including combustion science, aerosol science, epidemiology and toxicology.

## Competing interests

The authors declare that they have no competing interests.

## Authors' contributions

AKB planned and coordinated the study. All authors provided essential contributions to the manuscript and were involved in drafting the manuscript or revising it critically. All authors read and approved the final manuscript.

## Supplementary Material

Additional file 1 **Different types of wood combustion appliances**. The table provides a description of the four main types of wood combustion appliances mentioned in the text. Click here for file
