# Supplementary material for: Health effects of residential wood smoke particles: the importance of combustion conditions and physicochemical particle properties
Source: Part Fibre Toxicol. 2009 Nov 6;6:29. doi: 10.1186/1743-8977-6-29 (PMC2777846; doi:10.1186/1743-8977-6-29)
Supplement: Additional file 1 — Different types of wood combustion appliances. The table provides a description of the four main types of wood combustion appliances mentioned in the text. [file 1743-8977-6-29-S1.doc]

## Additional file 1 – Different types of wood combustion appliances

| Type of appliance | Explanation |
| --- | --- |
| Fireplace | is an [architectural](http://en.wikipedia.org/wiki/Architecture) structure designed to contain a fire for heating. The fire is contained in a fire pit and gas and [particulate](http://en.wikipedia.org/wiki/Particulate) exhaust are directed to escape through a [chimney](http://en.wikipedia.org/wiki/Chimney) |
| Stove | refers to the metal stoves commonly used in Scandinavia and central Europe. Generally these appliances consist of a closed metal fire chamber, a grate and an adjustable air control |
| Masonry heater | refers to heaters made of masonry products in combination with tiles, ceramic materials or soapstone where heat is stored in the large stone mass and slowly released to the surrounding room |
| Boiler | refers to heating systems where heat from the combustion chamber, or the exhaust gas, is transferred to water. Boilers are connected to a heat distribution system and may be installed with or without a water heat-accumulation tank |
